# Successful Closure of Postoperative Bronchopleural Fistula With Granulation After Submucosal Injection and Spreading of Fibrin Glue

**DOI:** 10.1002/ccr3.71103

**Published:** 2025-10-04

**Authors:** Satoshi Tanaka, Junji Uchida, Yasunobu Funakoshi, Kiyonobu Ueno

**Affiliations:** ^1^ Department of Respiratory Medicine Osaka General Medical Center Osaka Japan; ^2^ Department of Thoracic Surgery Osaka General Medical Center Osaka Japan

**Keywords:** bronchopleural fistula, fibrin glue, granulation, submucosal injection

## Abstract

In the treatment of bronchopleural fistulas, it is necessary to consider bronchoscopic intervention and surgery depending on the patient's condition and fistula size. Submucosal injection and spreading of fibrin glue under bronchoscopy may be useful methods for the closure of small, pinhole‐like fistulas that are several millimeters in size.

A 69‐year‐old man with a history of diabetes mellitus and heavy smoking underwent a thoracoscopic right lower lobectomy for lung cancer. After 16 days, the patient visited our outpatient clinic complaining of fever and dyspnea. The patient was immediately hospitalized with bacterial pneumonia in the right lower lobe (Figure 1A,B). The patient was treated with antimicrobial agents; however, chest radiography showed a worsening cystic shadow with niveau in the right lower lobe (Figure 2A), and computed tomography revealed a fistula at the suture site of the right lower lobe on Day 35 (Figure 2B). Bronchoscopy confirmed a pinhole‐shaped fistula at the suture site (Figure 3A). The patient was diagnosed with a bronchopleural fistula (BPF) and fistulous emphysema on Day 36. On Day 43, BPF closure was performed using a flexible bronchoscope. The procedure was performed under intubation using a flexible bronchoscope (P290; Olympus, Tokyo, Japan) and transbronchial aspiration needles (NA‐1C‐1 and NA‐2C‐1; Olympus). A total of 1.0 mL of each solution of fibrin glue (Beriplast P Combi‐Set Tissue adhesion; CSL Behring) was injected into the submucosa from the contralateral side of the fistula, respectively. Thereafter, the remaining 1.0 mL of each fibrin glue solution was spread onto the fistula. The procedure was performed in two phases on Days 43 and 50. On Day 50, the fistula was covered with granulation tissue (Figure 3B); however, an additional submucosal injection and application of fibrin glue were performed. On Day 63, the fistula was fully covered with granulation tissue (Figure 3C). Thereafter, there was no recurrence of pneumonia.

**FIGURE 1:**
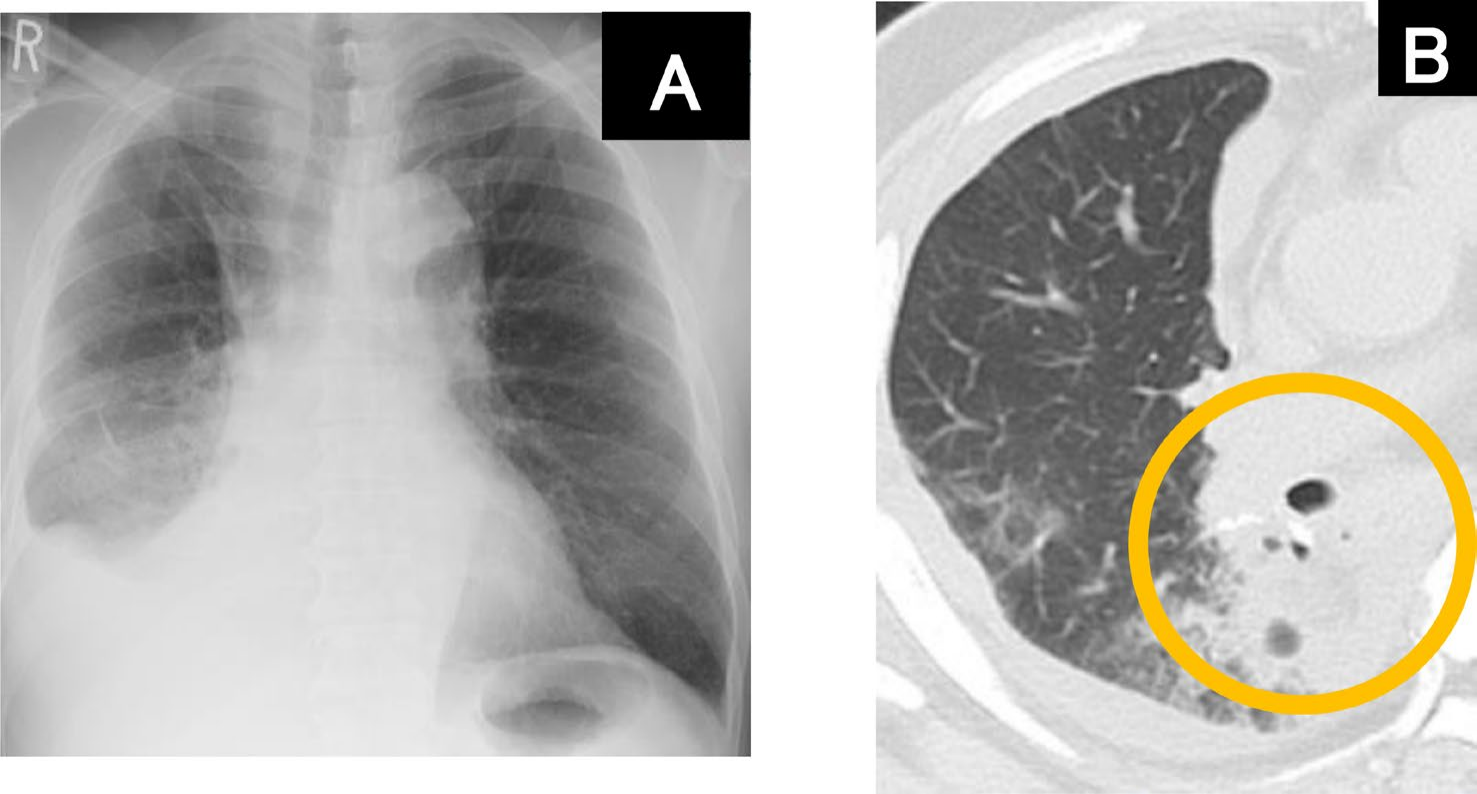
Radiological images on admission (on Day 16). (A) Chest radiograph showing decreased permeability and an infiltrating shadow in the right lower lobe. (B) Chest computed tomography showing an infiltrating shadow with air at the suture in the right lower lobe.

**FIGURE 2:**
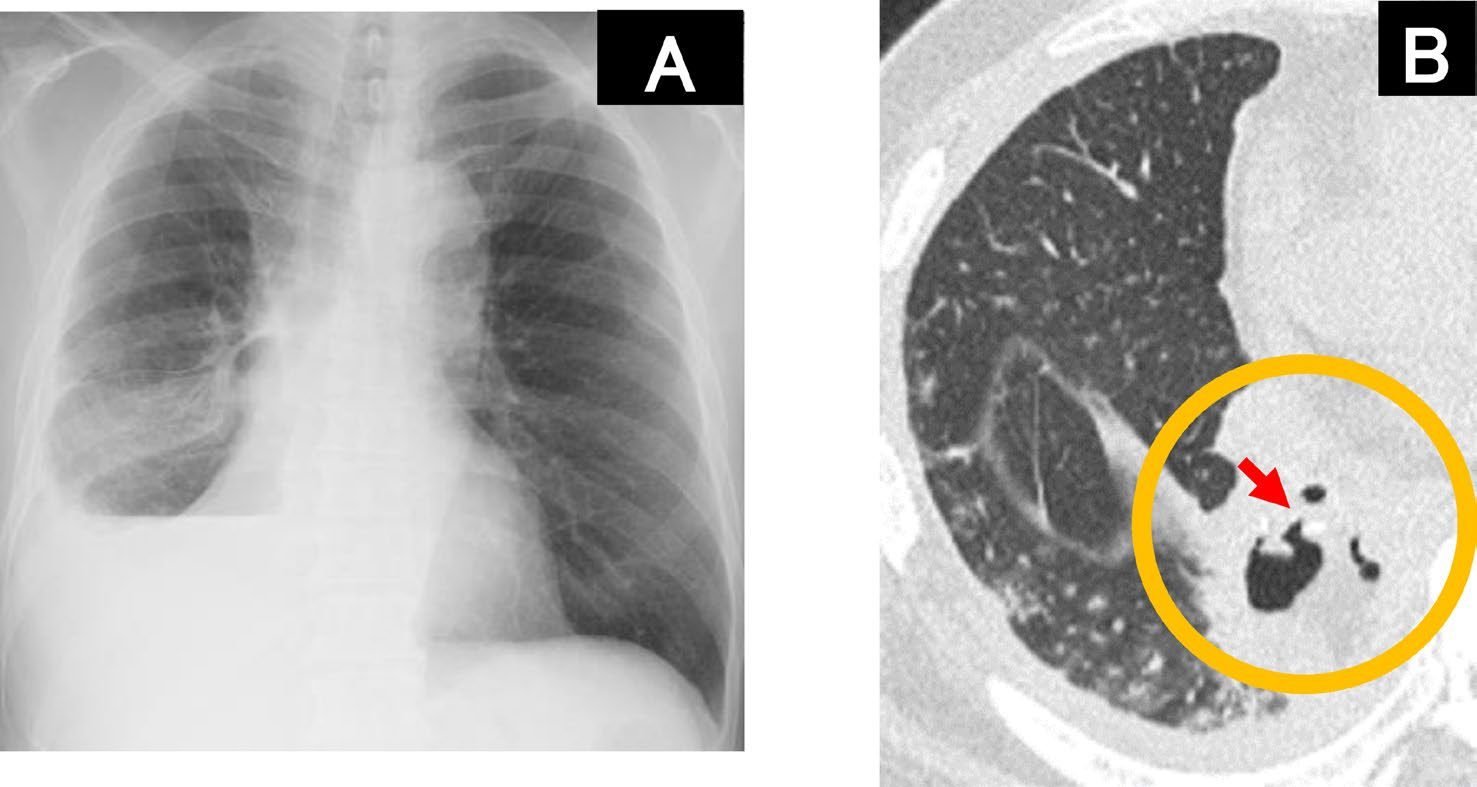
Radiological images on Day 35. (A) Chest radiograph shows a worsening cystic shadow with niveau in the right lower lobe. (B) Chest computed tomography showing an infiltrating shadow and fistula at the suture of the right lower lobe.

**FIGURE 3:**
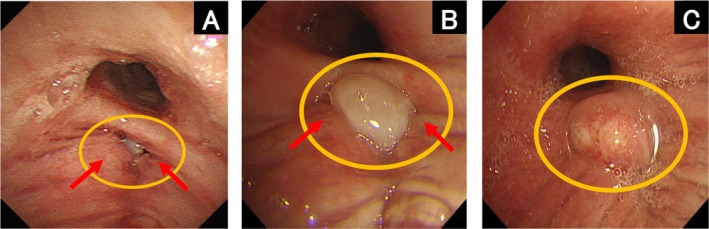
Bronchoscopic findings. (A) Flexible bronchoscopy showing a pinhole‐shaped fistula at the suture site (on Day 43). Fibrin glue is injected submucosally into the contralateral side of the fistula (red arrowhead). (B) Flexible bronchoscopy showing a fistula covered with granulation tissue (on Day 50). Fibrin glue is injected submucosally into the contralateral side of the fistula (red arrowhead). (C) Flexible bronchoscopy showing a fistula fully covered with granulation tissue (on Day 63).

BPF is mostly caused by lung resection surgery and has been reported to occur in 1.5%–4.5% of pneumonectomy cases and 0.5%–1% of lobectomy and sublobar resction cases [1]. There is no established strategy for the treatment of BPF, and it is necessary to consider bronchoscopic intervention and surgery depending on the patient's condition and fistula size. For BPFs with a diameter of > 8 mm, stents, coils, and occlusive devices are effective for fistula closure. However, sclerosing and plugging agents are effective for BPFs with diameters smaller than 8 mm [2]. Plugging agents include fibrin glue, cyanoacrylate glue, and polyvinyl alcohol sponges. In gastrointestinal endoscopy, submucosal injection of fibrin glue is a useful hemostatic method for gastrointestinal bleeding and a closure technique for perforation of the gastrointestinal tract [3]. There are few reports on the usefulness of submucosal injections of fibrin glue during bronchoscopy. We encountered a case of successful closure of a postoperative (BPF) with granulation after submucosal injection and spreading of fibrin glue under a flexible bronchoscope. This case is noteworthy in that granulation tissue formation could be observed over time using a flexible bronchoscope. Submucosal injection and spreading of fibrin glue under bronchoscopy may be useful methods for the closure of small pinhole‐like fistulas that are several millimeters in size.

## Author Contributions

**Satoshi Tanaka:** writing – original draft. **Junji Uchida:** supervision. **Yasunobu Funakoshi:** supervision. **Kiyonobu Ueno:** supervision.

## Ethics Statement

Approval code was not required because this study involved a case image (per the Institutional Ethics Committee of the Osaka General Medical Center).

## Consent

Written informed consent was obtained from the patient for the publication of this report in accordance with the journal's patient consent policy.

## Conflicts of Interest

The authors declare no conflicts of interest.

## Data Availability

The datasets used and/or analyzed during the current study are available from the corresponding author upon reasonable request.

## References

[1] M. Lois and M. Noppen , “Bronchopleural Fistulas: An Overview of the Problem With Special Focus on Endoscopic Management,” Chest 128, no. 6 (2005): 3955–3965, 10.1378/chest.128.6.3955. 16354867

[2] P. Zhao , “Progress Report on Interventional Treatment for Bronchopleural Fistula,” Emergency Medicine International 2023 (2023): 8615005, 10.1155/2023/8615055. PMC10310459 37398639

[3] P. Rutgeerts , E. Rauws , P. Wara , et al., “Randomized Trial of Single and Repeated Fibrin Glue Compared With Injection of Polidocanol in Treatment of Bleeding Peptic Ulcer,” Lancet 6, no. 9079 (1997): 692–696, 10.1016/s0140-6736(97)03233-9. 9291903

